# Substrate thiophosphorylation by Arabidopsis mitogen-activated protein kinases

**DOI:** 10.1186/s12870-016-0731-6

**Published:** 2016-02-24

**Authors:** Franz Leissing, Mika Nomoto, Marco Bocola, Ulrich Schwaneberg, Yasuomi Tada, Uwe Conrath, Gerold J. M. Beckers

**Affiliations:** Department of Plant Physiology, Aachen Biology and Biotechnology, RWTH Aachen University, Aachen, 52056 Germany; Division of Biological Science, Graduate School of Science, Nagoya University, Furo-cho, Chikusa-ku, Nagoya, Aichi 464-8602 Japan; Department of Biotechnology, Aachen Biology and Biotechnology, RWTH Aachen University, Aachen, 52056 Germany; The Center for Gene Research, Division of Biological Science, Nagoya University, Furo-cho, Chikusa-ku, Nagoya, Aichi 464-8602 Japan

**Keywords:** Mitogen-activated protein kinase, (thio-)phosphorylation, MPK3/4/6, *Arabidopsis thaliana*, Analog-sensitive kinase

## Abstract

**Background:**

Mitogen-activated protein kinase (MPK) cascades are important to cellular signaling in eukaryotes. They regulate growth, development and the response to environmental challenges. MPK cascades function via reversible phosphorylation of cascade components, MEKK, MEK, and MPK, but also by MPK substrate phosphorylation. Using mass spectrometry, we previously identified many in vivo MPK3 and MPK6 substrates in *Arabidopsis thaliana*, and we disclosed their phosphorylation sites.

**Results:**

We verified phosphorylation of several of our previously identified MPK3/6 substrates using a nonradioactive in vitro labeling assay. We engineered MPK3, MPK4, and MPK6 to accept bio-orthogonal ATPγS analogs for thiophosphorylating their appropriate substrate proteins. Subsequent alkylation of the thiophosphorylated amino acid residue(s) allows immunodetection using thiophosphate ester-specific antibodies. Site-directed mutagenesis of amino acids confirmed the protein substrates’ site-specific phosphorylation by MPK3 and MPK6. A combined assay with MPK3, MPK6, and MPK4 revealed substrate specificity of the individual kinases.

**Conclusion:**

Our work demonstrates that the in vitro-labeling assay represents an effective, specific and highly sensitive test for determining kinase-substrate relationships.

**Electronic supplementary material:**

The online version of this article (doi:10.1186/s12870-016-0731-6) contains supplementary material, which is available to authorized users.

## Background

Mitogen-activated protein kinase (MPK) cascades are conserved signaling modules in eukaryotes. They transduce external signals to intracellular responses via phosphorylation. MPK cascades contain three sequential types of protein kinases. These are MPKs, MPK-activating kinases (MKKs or MEKs), and MKK/MEK-activating kinases (MEKKs). Genetic and biochemical analyses identified distinct MEKK/MKK/MPK modules with overlapping functions in development, immunity, and abiotic stress responses [1–3]. In addition to MKKs, MPK activity is regulated by MPK phosphatases that dephosphorylate, and thereby inactivate their target MPKs. By now, the identity of many MPK substrates and the nature of the MPK-substrate interaction have remained elusive, especially in plants. Their disclosure is essential to understanding MEKK/MKK/MPK-mediated cell signaling.

Over the past decade, research on plant MPKs focused on the large-scale identification of MPK substrate proteins. For example, protein and peptide microarrays were probed with recombinant MPKs in the presence of radiolabeled ATP to identify novel MPK substrate proteins [4–6]. Another in vitro screen used a synthetic peptide library that was incubated with purified kinases before phosphorylation site identification by mass spectrometry [7]. Together, these screens revealed a large number of potential kinase-substrate relationships.

Novel protocols in phosphoproteomics enable the enrichment even of low-abundant MPK substrate proteins thus making them accessible to mass spectrometry [8, 9]. These protocols describe dual enrichment strategies that include the enrichment of phosphoproteins by Al(OH)_3_-based metal-oxide affinity chromatography (MOAC). The Al(OH)_3_-based MOAC is either preceded by an ammonium-sulfate prefractionation step [9], or followed by a specific enrichment of phosphopeptides using TiO_2_. We referred to the latter approach as tandem MOAC [8]. The tandem approach enables the direct recording of site-specific phosphorylation of several known and many unknown substrate candidate proteins of MPK3 and MPK6 in *Arabidopsis thaliana*.

Here, we verify selected, previously in vivo identified MPK substrate proteins of Arabidopsis using a novel nonradioactive in vitro labeling assay for determining plant MPK substrate phosphorylation [8]. We use ATPγS analogs that cannot enter the ATP-binding pocket of a wild-type kinase, but can do so when the kinase’s ATP-binding pocket is enlarged. These so-called analog-sensitive (AS) kinases use bulky ATPγS analogs as cofactors during catalysis. We engineered the proline-directed serine/threonine kinases MPK3, MPK4, and MPK6 of Arabidopsis by mutating the gatekeeping amino acid in the ATP-binding pocket of the appropriate kinases. The mutation enlarges the ATP-binding pocket thus allowing the AS kinase to catalyze thiophosphorylation of its substrate proteins. Subsequent alkylation of thiophosphorylated serine and/or threonine residues provides a semisynthetic epitope for a monoclonal thiophosphate ester-specific (anti-TPE) antibody [10]. In addition to verifying previously identified in vivo MPK3/6 substrates in Arabidopsis, we demonstrate that these MPK3/6 substrates are poor substrates for the closely related Arabidopsis MPK4 [5, 8].

## Results

### Arabidopsis MPKs use ATPγS to thiophosphorylate myelin basic protein

**Fig. 1:**
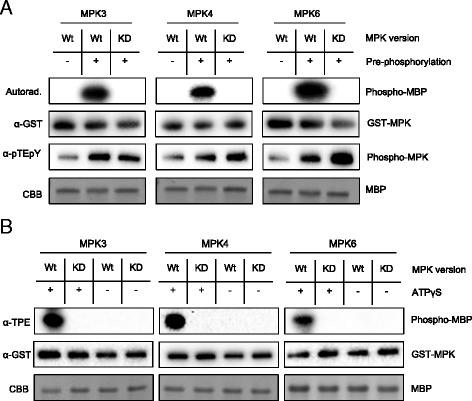
MBP Phosphorylation and Thiophosphorylation by MPK3/4/6. **a** Kinase activity assay of purified wild-type (Wt) and kinase-death (KD) forms of MPK3/4/6 with (+) and without (-) pre-phosphorylation by upstream MKKs. **b** In vitro thiophosphorylation of MBP by Wt and KD forms of MPK3/4/6 in the presence (+) or absence (-) of ATPγS. CBB: Coomassie Brilliant Blue

In conventional in vitro kinase assays, a kinase and its substrate are incubated in the presence of [γ-^32^P] or [γ-^33^P]-labeled ATP. Upon incubation, ^32^P/^33^P-radiolabeled substrate is being detected as a measure for kinase activity or suitability of a protein or peptide to serve as a substrate for the kinase in question. To determine the in vitro activity of Arabidopsis MPK3, MPK4, and MPK6 towards selected proteinaceous candidate substrates in the absence of radioactive labeling, we expressed MPK3, MPK4, and MPK6 as GST-fusions in *Escherichia coli*. Fusion proteins were purified and incubated, in catalyzing conditions, in the presence of the generic MPK substrate myelin basic protein (MBP) [11]. MPK activity was ensured by phosphorylation of column-bound GST-MPKs (before elution) using purified, constitutively active versions of upstream MKKs in the presence of ATP. GST-MPK3 and GST-MPK6 were activated with constitutively active MKK4 and MKK5 (subsequently referred to as MKK4^DD^ and MKK5^DD^), whereas column-bound GST-MPK4 was activated by MKK1^DD^ and MKK2^DD^. Before elution of the GST-fused MPKs, the column was extensively washed with buffer to remove any residual MKK. As controls we used equally treated kinase-death versions of MPK3, MPK4, and MPK6 (subsequently referred to as MPK3^KD^, MPK4^KD^, and MPK6^KD^). Pre-incubation of MPK3^KD^/4^KD^/6^KD^ with their appropriate upstream MKK^DD^s induced dual phosphorylation of the TEY motif in the activation loop of kinases, but did not stimulate MPK3^KD^/4^KD^/6^KD^ to phosphorylate MBP (Fig. 1a). In contrast, on-column pre-incubation of wild-type MPKs with their upstream MKK^DD^s not only enhanced dual phosphorylation of the MPK’s TEY motif, but it also strongly induced MPK3/4/6 activity (Fig. 1a).

Next, we asked whether MPK3/4/6 accept ATPγS as a cofactor to thiophosphorylate their generic substrate MBP. We also wondered whether a commercial anti-TPE antibody can be used to detect thiophosphorylated substrate proteins of MPK3/4/6. To answer these questions, pre-phosphorylated wild-type and kinase-death versions of MPK3/4/6 were incubated with MBP in the presence or absence of ATPγS. After adding *p*-nitrobenzyl mesylate (PNBM) for alkylating the potential thiophosphoryl group on MBP, the reaction mixture was subjected to standard western blotting analysis and immunodetection with anti-TPE antibody. No signal was detected in control reactions without ATPγS, or when kinase-death MPKs were used in the assay with ATPγS (Fig. 1b). However, the anti-TPE antibody cross-reacted with MBP in reaction mixtures containing only active wild-type MPK3/4/6 and ATPγS (Fig. 1b). This result strongly suggested that MPK3/4/6 all accept ATPγS as a phosphoryldonor to thiophosporylate MBP and supposedly also other substrate proteins. It also disclosed that the commercial anti-TPE antibody can be used to detect thiophosphoryl groups on MPK3/4/6 substrate proteins (Fig. 1b).

### Engineered Arabidopsis AS-MPKs use N^6^-benzyl-ATPγS as cofactor

**Fig. 2:**
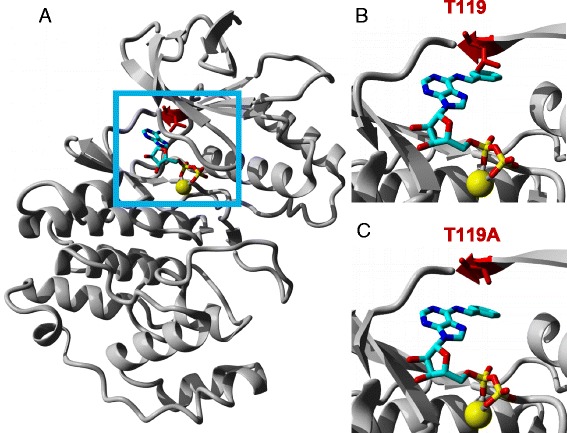
Model of Wild-type and Mutant Variants of MPK3 with Bn-ADP. **a** Ribbon diagram of wild-type MPK3 with its ligands ADP (shown in stick mode and colored by atom type) and Mg^2+^ (yellow). The backbone and amino acid side chain of T119 in MPK3 are highlighted in red and shown in stick mode. Blue outlined rectangle highlights the ATP-binding site of MPK3. **b** Close-up of Bn-ADP modeled into the ATP-binding site of wild-type MPK3. **c** Same as in (**b**) but with MPK3^T119A^

Next we asked whether AS-MPK3/4/6 would accept synthetic N^6^-benzyl-ATPγS (Bn-ATPγS) as a thiophosphate donor to exclude thiophosphorylation of substrates by contaminating kinases and to also prevent substrates from potential autophosphorylation. Earlier studies identified two tyrosine residues (Y124 in MPK4 and Y144 in MPK6) as the gatekeeper amino acid in these two kinases [12, 13]. To identify the gatekeeping residue in MPK3, we built a 3D atomic structure of MPK3 and Bn-ADP (Fig. 2a). The model predicts an atomic clash of the large threonine-119 (T119) amino acid residue in MPK3 with the bulky side chain of Bn-ADP (Fig. 2b). Mutation of T119 to a smaller amino acid, such as alanine (A), would probably allow Bn-ATP to access the ATP-binding site of MPK3 (Fig. 2c). Thus, T119 seems to be the gatekeeper amino acid residue in Arabidopsis MPK3.

**Fig. 3:**
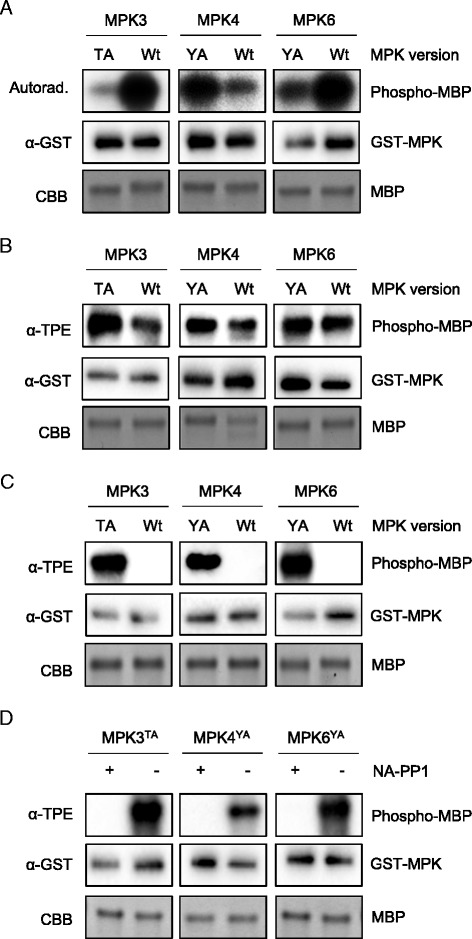
Activity of AS-MPKs and Inhibition by NA-PP1. **a** Activity assay of wild-type and AS variants of MPK3/4/6 with MBP as substrate and [γ-^32^P] ATP as cofactor. **b** Same as in (A) but with ATPγS as cofactor in the substrate labeling reaction. **c** Same as in (B) but with Bn-ATPγS as cofactor. **d** Activity assay of AS-MPK3/4/6 with MBP as substrate and Bn-ATPγS as cofactor in the presence (+) or absence (-) of NA-PP1

To test our *in silico* model (Fig. 2) in vitro, we purified GST-MPK3^T119A^ that was expressed in *E.coli*. We also purified a mutant version of AS-MPK4 (GST-MPK4^Y124A^) and AS-MPK6 (GST-MPK6^Y144A^) that we had expressed in *E. coli*. First, the relative activity of these AS kinases to phosphorylate MBP was compared to the activity of their appropriate wild-type versions in the presence of either [γ-^32^P] ATP (Fig. 3a) or ATPγS (Fig. 3b). Mutation of Y124 to alanine did not affect (Fig. 3b) MPK4’s ability to use [γ-^32^P] ATP (Fig. 3a) or ATPγS (Fig. 3b). MPK6^Y144A^ phosphorylated MBP to an about same (Fig. 3b) or slightly lower extent (Fig. 3a) than the MPK6 wild-type protein. MPK3^T119A^ also catalyzed MBP phosphorylation which was lower (Fig. 3a) or somewhat higher (Fig. 3b) than it was with the wild type.

In another set of experiments, the ability of AS-MPK3/4/6 to use Bn-ATPγS as a cofactor during catalysis was tested in in vitro substrate labeling assays (Fig. 3c). Wild-type MPK3/4/6 did not use Bn-ATPγS as a thiophosphate donor, whereas all AS kinases used the bio-orthogonal Bn-ATPγS analog to thiophosphorylate MBP (Fig. 3c).

AS-MPKs can be specifically inhibited by purine analogs that do not affect the activity of wild-type kinases. For example, previous studies revealed that 4-amino-1-tert-butyl-3-(1′-naphthyl)pyrazolo[3,4-d]pyrimidine (NA-PP1) specifically inhibits AS kinases but not their appropriate wild-type kinase because its bulkier side chain prevents NA-PP1 from accessing the ATP-binding pocket of wild-type kinases [12, 13]. Consistent with this AS-MPK3/4/6 seem to be sensitive to NA-PP1 because NA-PP1 addition to the substrate labeling reaction completely abolished MPK3^T119A^, MPK4^Y124A^, and MPK6^Y144A^ activity (Fig. 3d).

### Site-specific phosphorylation of MPK targets by in vitro substrate labeling

**Fig. 4:**
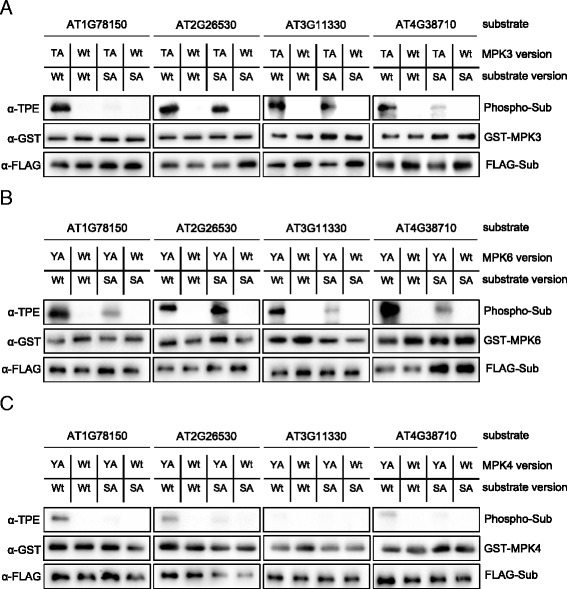
In vitro Labeling Assay of Wild-type and Mutated MPK Substrates. **a** Thiophosphorylation assays of native (Wt) and phosphosite mutant (SA) forms of FLAG-tagged MPK substrates AT1G78150, AT2G26530, AT3G11330 and AT4G38710 in the presence of Bn-ATPγS as cofactor and using either AS-MPK3 or Wt-MPK3 as a negative control. **b** Same as in (A) but using AS-MPK6 and Wt-MPK6. **c** Same as in (**a**) but using AS-MPK4 and Wt-MPK4

Next, we used in vitro substrate labeling reactions with active AS-MPK3/4/6 to verify previously identified in vivo MPK3/6 substrates and their target phosphorylation sites. We randomly selected four MPK3/6-specific in vivo substrates which we identified by tandem MOAC in previous work [8]: two proteins of unknown function (AT2G26530 and AT1G78150), a putative translation initiation factor (AT4G38710), and PIRL9, a member of the Plant Intracellular Ras group-related leucine-rich repeat (LRR) proteins (AT3G11330). In addition to the wild-type version of proteins, we cloned phosphosite mutants in which the previously recorded phosphorylated serine and/or threonine residues were mutated to alanine. FLAG-tagged wild-type and mutant proteins were immunoprecipitated with anti-FLAG agarose resin after successful in vitro translation in wheat germ extracts. While bound to the affinity gel, proteins were incubated with active AS-MPK3/4/6 or the appropriate wild-type form of kinase in the presence of Bn-ATPγS. As shown in Fig. 4, wild-type AT1G78150, AT2G26530, and AT4G38710 were phosphorylated by all three MPKs. However, in contrast to MPK3/6, these proteins seem to be only weakly phosphorylated by MPK4^Y124A^ (Fig. 4c). AT3G11330 is a good substrate of MPK3/6 but not phosphorylated by MPK4 (Fig. 4a–c). In most cases, mutation of previously identified phosphosites in the investigated proteins strongly reduced the extent of phosphorylation by MPK3/4/6 in vitro. These findings indicate that the previously identified serine/threonine residues are specifically targeted by MPK3/6 and to a lesser extent by MPK4.

**Fig. 5:**
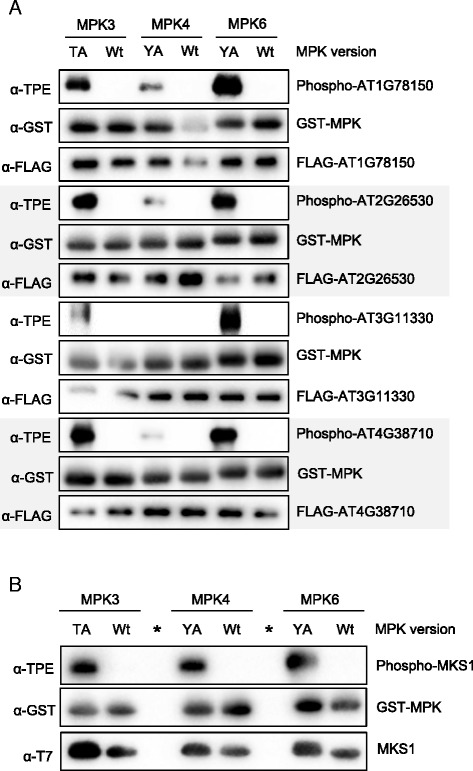
Substrate Preference of MPK3/4/6. **a** Thiophosphorylation assays of native, FLAG-tagged AT1G78150, AT2G26530, AT3G11330 and AT4G38710 in the presence of Bn-ATPγS as cofactor and using either AS-MPK3/4/6 or Wt-MPK3/4/6 as a negative control. **b** Same as in (**a**) but using *E. coli* expressed T7-tagged MKS1 as a substrate. Sample wells that were left empty are indicated by an asterisk (*)

The results in Fig. 4 indicated that AT1G78150, AT2G26530, AT3G11330, and AT4G38710 are good substrates for MPK3/6 but are not, or only weakly phosphorylated by MPK4. To directly compare the potential of MPK3/4/6 to phosphorylate the four proteins, we repeated the substrate labeling reactions with all three MPKs (Fig. 5a). We tested the wild-type version of AT1G78150, AT2G26530, AT3G11330, and AT4G38710 and loaded the samples of each of these proteins in combination with MPK3/4/6 on a separate gel. The results of this experiment support the previous finding arguing that the assayed proteins represent good substrates of MPK3/6, but are marginally phosphorylated by MPK4. To exclude the possibility that the lower level of phosphorylation of these MPK3/6 substrates by MPK4 is due to a lower in vitro activity of MPK4, we expressed and purified the known MPK4 substrate MAP kinase substrate 1 (MKS1) from *E. coli*, and found that MKS1 was equally well phosphorylated by MPK3/6 and 4 (Fig. 5b) [14]. Together, these data verify AT1G78150, AT2G26530, AT3G11330, and AT4G38710 as substrate proteins of MPK3/6, and they suggest specificity in the in vitro substrate-labeling reactions.

### Specificity of the in vitro substrate labeling assay

**Fig. 6:**
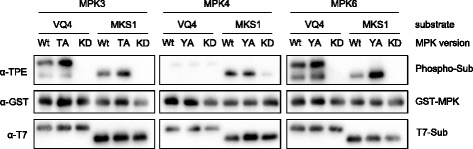
Comparison of Substrate Specificity of Wild-type and AS-MPKs. Thiophosphorylation assays of T7-tagged VQ4 and MKS1 in the presence of ATPγS as cofactor and using wild-type, AS-MPK, and KD-MPK versions of MPK3/4/6

**Fig. 7:**
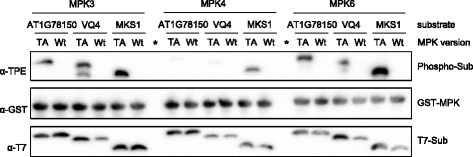
Specificity of Substrate Phosphorylation by AS-MPK3/4/6. Thiophosphorylation assays of T7-tagged AT1G78150, VQ4 and MKS1 in the presence of Bn-ATPγS as cofactor and using either AS-MPK3/4/6 or Wt-MPK3/4/6 as negative controls. Sample wells that were left empty are indicated by an asterisk (*)

Arabidopsis MPKs are not only related in sequence but they also share substrates [4, 5]. Substrate overlaps have been reported mainly for MPK3 and MPK6, but also for MPK3 and MPK4. To analyze the specificity of the in vitro substrate labelling reaction, we first examined whether the mutation of the gatekeeper amino acids of MPK3/4/6 to alanine leads to a change in substrate selectivity. Therefore we compared substrate specificity of wild-type versus AS-MPKs in in vitro labelling reactions containing ATPγS. As MPK-substrates we chose two members of the VQ-motif-containing protein family, the MPK3/6 substrate VQ4, and the well described MPK4-specific substrate MKS1 (also known as VQ21) [8, 14, 15]. Whereas AS-MPK4 and wild-type MPK4 could only markedly thiophosphorylate MKS1, both VQ4 and MKS1 were thiophosphorylated by wild-type and mutated MPK3 and MPK6 (Fig. 6). Thus these results indicate that mutation of the gatekeeper amino acid to alanine does not influence substrate specificity of MPK3/4/6. In a final experiment, to compare the substrate preference of AS-MPK3, AS-MPK4, and AS-MPK6 using bio-orthogonal Bn-ATPγS, we again tested VQ4 and MKS1 but also included the MPK3/6-specific substrate AT1G78150 [8, 14]. The three MPK substrates were expressed and purified from *E. coli* as native His_6_-tagged fusion proteins with an additional N-terminal T7 epitope for immunodetection. To evaluate the substrate preference of AS-MPK3, AS-MPK4, and AS-MPK6 we performed in vitro labeling assays using equal amounts of the above mentioned protein substrates. The samples were loaded on a same gel to allow for the direct comparison of signal intensities. Consistent with the results in Figs. 4 and 5, the unknown protein AT1G78150 was well phosphorylated by MPK3 and MPK6 and only weakly by MPK4 (Fig. 7). Likewise, VQ4, a specific MPK3/6 substrate protein [8] also was only weakly phosphorylated by MPK4 (Fig. 7). In contrast, the known MPK4 substrate MKS1 was not only phosphorylated by MPK4, as previously reported [14, 16], but is equally well phosphorylated by MPK3/6 (Figs. 5b and 7). This result shows that by including known substrates of the three major plant MAP kinases as positive controls on the same gel, the in vitro labeling reactions enable assessment of the specificity of kinase-substrate interactions.

## Discussion and conclusions 

Traditional in vitro kinase assays employ kinases, their substrates and γ-^32^P or γ-^33^P-labeled ATP. Nowadays, the radiolabeled nucleotide can be substituted by non-radioactive ATPγS resulting in thiophosphorylation of the kinase substrate. Following alkylation with PNBM, the thiophosphorylated substrate can be detected by western blotting analysis and immunodetection with an anti-TPE antibody [10]. Specific substrate labeling is achieved by engineering the kinase of interest to accept bulky ATPγS analogs that, because of steric hindrance cannot be used by naive kinases. Until now, this approach for studying kinase-substrate interactions in vitro has not been applied in plant biology research. We used the approach to show that AS-MPK3/4/6 are able to use Bn-ATPγS to thiophosphorylate substrates, and that there is specificity in these in vitro reactions. In addition, we validated the phosphorylation of previously identified in vivo MPK3/6 protein substrates, and we demonstrated that these substrates are rather poor substrates for MPK4.

The substrate thiophosphorylation assay is simple, effective, and has high sensitivity. It does not need work with hazardous material or problematic waste disposal. Another advantage of using AS kinases and bio-orthogonal ATP-analogs is the specificity of the kinase-substrate interaction. This is of particular relevance when (i) the kinase reaction is performed with substrate proteins of suboptimal purity, (ii) when an additional upstream activating kinase is required for the reaction, or (iii) if the putative substrate is a protein with kinase activity. The latter is true, for example, for the MPK3/6-specific substrate PIRL9 (AT3G11330) (Figs. 4 and 5) [17]. Using AS kinases and ATP analogs thus avoids the undesired detection of such autophosphorylation and/or phosphorylation by contaminating kinases in the reaction mixture.

Until now, AS kinases have been widely exploited when studying cell signaling in yeast and mammals [10, 18]. In Arabidopsis, AS-MPK4 or AS-MPK6 were used to genetically complement the *mpk4* or *mpk6* mutant [12, 13]. The authors mutated the gatekeeper amino acid to glycine. The exchange leads to a specific inhibition of the kinases in vivo upon application of NA-PP1. In the present work, we for the first time applied AS-MPK3/4/6 in substrate labeling reactions. Since glycine lacks an amino acid side chain, it often causes a sharp turn of the polypeptide backbone [19] and, thus, may result in a collapse of the ATP-binding pocket and the associated loss of kinase activity. By contrast, introducing an alanine at the gatekeeper position not only preserved the enzyme activity (Fig. 3), and substrate specificity (Figs. 6 and 7) of AS-MPK3/4/6, but also maintained the possibility to block their activity by binding of NA-PP1 in the enlarged active site pocket (Fig. 3).

Our previous work identified in vivo MPK target proteins using tandem-MOAC combined with dexamethasone-inducible expression of a constitutively active tobacco MPK-kinase (*Nt*MEK2^DD^) in Arabidopsis [8]. *Nt*MEK2 phosphorylates and thus activates *Arabidopsis* MPK3 and MPK6 [20]. However, we cannot exclude that the activity of other MPKs is affected as well in these plants. Here, we validated in vitro that AT2G26530, AT1G78150, AT4G38710, and AT3G11330 are phosphorylated by MPK3/6, but are poor substrates for *Arabidopsis* MPK4 (Figs. 4 and 5). Except for AT2G26530, knocking out the phosphorylation-targeted residue by site-directed mutagenesis of the serine or threonine amino acid within the serine-proline or threonine-proline dipeptide motif resulted in a major decrease in the phosphorylation of these proteins (Fig. 4). However, besides the identified phosphorylation site of AT2G26530, the protein contains eight additional putative MPK phosphorylation sites suggesting that MPK3/6 might target additional phosphosites in AT2G26530 (Additional file 1: Figure S1). Together, these findings disclose the power of our tandem-MOAC analyses and the high confidence of phosphosite localization probability. To assess substrate preferences, we not only assayed MPK3/4/6 phosphorylation of each substrate independently (Figs. 4 and 5), but we also directly compared phosphorylation of several substrates by any of these MPKs (Fig. 7). Consistent with results in other laboratories, MKS1 is a good substrate for MPK4 (Figs. 5, 6 and 7) [14, 21]. However, based on our data we conclude that MKS1 also is a good substrate for MPK3/6, which contrast recent reports by Sörensson et al. (2012) and Pecher et al. (2014) [16, 21].

## Methods

### Cloning and site-directed mutagenesis

Coding regions of *MPK3, MPK4*, and *MPK6* were amplified by PCR, ligated in frame into pGEX5x-3 vector (GE Healthcare) and sequenced. *MKK1, MKK2, MKK4, MKK5, MKS1, VQ4* and *AT1G78150.1* were cloned into pET_λ_HIS [22] using restriction enzymes listed in Additional file 1: Table S1 and transformed into *E. coli* BL21. Coding regions of the MPK substrates *AT2G26530, AT1G78150, AT4G38710*, and *AT3G11330* were amplified by PCR and cloned into pJET1.2 (Thermo Scientific). Site-directed mutagenesis (Additional file 1: Table S2) was performed either by double joint PCR as described [23] or using the QuickChangeII site-directed mutagenesis kit (Stratagene).

### Protein expression and purification

For recombinant protein expression 2.5 mL of an *E. coli* overnight culture was diluted in 250 mL LB medium. The culture was grown at 37 °C to an OD_600_ of 0.8, supplemented with 1 mM IPTG and incubated at 28 °C for another 3 h. Cells were harvested by centrifugation at 4000 × g and 4 °C for 15 min and stored at -80 °C until further processing. Proteins were purified either using GSTrap FF (GE Healthcare) columns for purification of GST-tagged proteins or Ni^2+^-NTA columns (Qiagen) for the purification of His-tagged proteins. For phosphorylation of GST-MPKs by their respective, constitutively active MKK^DD^ (MKK4/5^DD^ for MPK3/6; MKK1/2^DD^ for MPK4), on-column immobilized GST-MPK was incubated for 2 h with 1 μg purified His-tagged MKK^DD^ in kinase buffer (50 mM Tris-HCl pH 7.5, 10 mM MgCl_2_, 1 mM DTT, 1 mM ATP). After an additional washing step, the phosphorylated GST-MPK was eluted according to manufacturer’s instructions. Protein concentrations were determined with the Bradford protein assay kit (Bio-Rad) using BSA as the standard.

### In vitro transcription and translation

FLAG-tagged AT1G78150, AT2G26530, AT3G11330 and AT4G38710 were synthesized using the IN VITRO Transcription/Translation Reagents kit following the manufacturer’s instructions (BioSieg).

### Radioactive kinase activity assay

Radioactive kinase assays were performed as described [20]. Briefly, 100 ng recombinant active GST-MPK3, 4 or 6 were mixed with 3 μg MBP in kinase reaction buffer (50 mM Tris-HCl pH 7.5, 10 mM MgCl_2_, 1 mM DTT) with 25 μM ATP and [γ-^32^P]-ATP (1 μCi per reaction). After 30 mins, the reaction was stopped by adding SDS loading buffer. The phosphorylation of MBP was visualized after SDS-PAGE by autoradiography. Loading of MBP was visualized by PageBlue™ Protein Staining Solution (Thermo Scientific).

### Substrate labeling reactions

Substrate labeling reactions were performed as described [10]. In brief, 100 ng recombinant active GST-MPKs were mixed either with 3 μg MBP, 1 μg recombinant T7-tagged MPK substrate, or 10 μL immunoprecipitated FLAG-tagged substrates in kinase reaction including either 1 mM ATPγS (Sigma Aldrich) or 1 mM N6-Bn-ATPγS (Biolog). For immunocomplex substrate labeling, in vitro translated FLAG-tagged proteins were immunopreciptated with 40 μL EZview^TM^ Red ANTI-FLAG M2 affinity Gel (Sigma Aldrich) according to manufacturer’s instructions. While still binding the FLAG-tagged substrate, the affinity gel was washed twice with kinase buffer and the resin was resuspended in 40 μL kinase buffer. For each substrate labeling reaction 10 μL of the bead suspension was used. The reaction was stopped by adding 20 mM EDTA after 1 h and the thiophosphorylated substrate was alkylated with 2.5 mM PNBM (Abcam) in 5 % (v/v) DMSO for 2 h. The alkylation reaction was stopped by adding SDS loading buffer. Samples were subjected to SDS-PAGE, transferred to a nitrocellulose membrane (Carl Roth), and used for immunodetection as described [24]. Primary rabbit antibodies against the thiophosphatester (α-TPE, Abcam) were used for detection of thiophosphorylation. The anti-phospho-p44/42 MPK (Thr202/Tyr204) antibody, which detects doubly phosphorylated MPK3/4/6, was from New England Biolabs. Rabbit anti-T7 (Cell Signaling Technologies) and mouse monoclonal anti-FLAG M2 (Sigma Aldrich) epitope antibodies served as loading control of FLAG-tagged and T7-tagged MPK substrates. Rabbit anti-GST (Cell Signaling Technologies) antibodies were used to check equal amounts of kinase in each reaction. Antigen-antibody complexes were detected with horseradish peroxidase-coupled anti-rabbit or anti-mouse secondary antibodies (Cell Signaling Technologies) followed by chemiluminescence detection with Luminata Crecendo HRP substrate (Millipore). Using independent protein preparations, all substrate labelling reactions were repeated at least twice with similar results.

### Bioinformatics

The homology model of MPK3 was generated with HHpred 2.0 and MODELLER [25, 26] based on the X-ray crystal structure of human MPK7/ERK5, PDB: 4ic7, sequence identity of 51 % and similarity of 0.890; human MPK12, PDB: 1 cm8, sequence identity of 41 % and similarity of 0.818; yeast FUS3, PDB: 2b9h, sequence identity of 50 % and similarity of 0.922; human MPK8, PDB: 2xrw, sequence identity of 41 % and similarity of 0.717 and human, CDK7, PDB: 1ua2, sequence identity of 41 % and similarity of 0.609. The model structure of MPK3 was overlayed by MUSTANG [27] with the ADP and Mg2+ bound to the FUS3 structure (PDB: 2b9h) using YASARA structure version 14.7.17 [28] and the initial binding mode of the Mg^2+^/ADP cofactor was introduced into the apo-model structure of MPK3. Based on this initial MPK3 Mg^2+^/ADP bound model, the binding mode of the bulky N6-benzyl-ATP was manually build and energy minimized using YASARA [29]. To remove atomic clashes and correct the covalent geometry, first a short steepest descent minimization was performed. After removal of conformational stress the procedure continued by simulated annealing (timestep 2 fs, atom velocities scaled down by 0.9 every 10th step) until convergence was reached, i.e. the energy improved by less than 0.05 kJ/mol per atom during 200 steps. We applied the AMBER03 [30] force field for protein residues and the general amber force field GAFF [31] utilizing AM1BCC [32] calculated partial charges and a force cutoff of 0.786 Å and particle mesh Ewald [33] for exact treatment of long range electrostatics using periodic boundary conditions. The same procedure was performed with the active site mutation T119A.

## Conclusion

Our data show that the in vitro-labeling assay represents an effective, specific and highly sensitive test for determining kinase-substrate relationships using Arabidopsis MPKs. By applying the analog-sensitive MPK3 and MPK6 we confirm previously identified in vivo phosphorylation of MPK3/6 substrates and demonstrate that these substrates are poor targets for the closely related Arabidopsis MPK4.

### Availability of data and materials

All supporting data can be found within the manuscript and its additional files.

### Highlight

We describe a novel nonradioactive in vitro labeling assay for determining plant MPK protein substrate phosphorylation. The assay is effective, specific, and highly sensitive for determining kinase-substrate relationships.

## Additional file

Additional file 1: Figure S1. Protein sequences of selected MPK3/6 substrates. **Table S1.** List of primers used for cloning. **Table S2.** List of primers used for site-directed mutagenesis. (DOCX 21 kb)
