# Supplementary material for: Substrate thiophosphorylation by Arabidopsis mitogen-activated protein kinases
Source: BMC Plant Biol. 2016 Feb 24;16:48. doi: 10.1186/s12870-016-0731-6 (PMC4765092; doi:10.1186/s12870-016-0731-6)
Supplement: Additional file 1: Figure S1. — Protein sequences of selected MPK3/6 substrates. Table S1. List of primers used for cloning. Table S2. List of primers used for site-directed mutagenesis. (DOCX 21 kb) [file 12870_2016_731_MOESM1_ESM.docx]

*BMC Plant Biology*

Substrate thiophosphorylation by Arabidopsis mitogen-activated protein kinases

Leissing F, Nomoto M, Bocola M, Schwaneberg U, Tada Y, Conrath U, Beckers GJM.

Supplementary Data File

> AT2G26530

MEVMSLTAPSSPRQLSGCFLSAPTSPRRFNEFYREFEEAATRNFSDRLTVPFDWEETPG(ph)TPRKITNDDDDDIDFAFEIGGKLETTSLFAEELFDGGKIKPLKPPPYLQLDHHHQPQILSPRSPRSPIAHGKNIIRKAFSPRKKPDNVDPFEVAMDKARNGLGEERGRGRRQNSGRRVARSLSPFRVSAYPWEEQEQEQEQEQRDVQEQRKGTLSSIPSTSSSACVSCKSSSSKKWRLKDFLLFRSASEGRARHNKDSVKTFTSLFRKQEDTKNSSSRGRGSSSVSAHEFHYMSKKAETKDLKKKTFLPYMQIGRFAF

> AT1G78150

MERSTPVRKPHTSTADLLTWSEVPPPD(ph)SPSSASRSAVRSHQPSDGISKVVFGGQVTDEEVESLNRRILDDAFDSFMRLVIYTNVKTCENVYDVIRKPCSEHKMKEITGSGIFSRNEKDDASEPLPVYQQAVNGISQISFGEEENLSPKKPATVPEVAKQRELSGTMENESANKLQKQLSDAKYKEISGQNIFAPPPEIKPRSGTNRALALKDNFNLGAESQTAEEDSSVKTAKKIYDKKFAELSGNDIFKGDAASSNVEKHLSQAKLKEIGGNNIFADGKVEARDYLGGVRKPPGGETSIALV

> AT4G38710

MAAAVSSVWAKPGAWALEAEEHEAELKQQPSPTNQKSSAEDSSDFPSLAAAATTKTKKKKGQTISLAEFATYGTAKAKPAPQTERLTQAELVALPTGPRERSAEELDRSKLGGGFRSYGGGRYGDESSSSRWGSSRVSEDGERRGGGFNRDREPSRDSGPSRADEDDNWAAAKKPISGNGFERRERGSGGGFFESQSQSKADEVDSWVSTKPSEPRRFVSSNGGGGDRFEKRGSFESLSRNRDSQYGGGGGSESDTWGRRREESGAANGSPPPSGGSRPRLVLQPRTLPVAVVEVVKPE(ph)SPVLVIVEKPKGANPFGNARPREEVLAEKGQDWKEIDEKLEAEKLKDIAAAMEKPNEKSTGKMGFGLGNGRKDEERIERSWRKSFSLHSYMEVDVLNTEHSEEDAQEEEPAVEGAKKEETEDKPAVEEAKKEETEGEQAVEEAKKEETGGEPAVEEAKKEETEDKI

> AT3G11330

MAAEPNPKNFPVLSYVLARLPSFTAKSPSSSVPPFDIEQPPPSSSSSSIEIVTQMPHLTQPDVLASMTSAISDVAETRSILRTLGPRPDHESVDKARAKLSEIESFLSESFEDIALTDAAAKDEKRRQEMDQEKTWCESILKLDEVHASYEKLLKEAEERLVRIYESAEKNAAEDEENVAAVEVNEEVVGILQHASANPVDRVDLSGRKLRLLPEAFGRIQGLLVLNLSNNKLESIPDSIAGLHSLVELDVSTNSLETLPDSIGLLSKLKILNVSTNKLTSLPDSICRCGSLVILDVSFNRLTYLPTNIGPELVNLEKLLVQYNKIRSFPTSIGEMRSLKHLDAHFNELNGLPDSFVLLTNLEYLNLSSNFSDLKDLPFSFGELISLQELDLSNNQIHALPDTFGTLDSLTKLNVDQNPLVVPPEEVVKEGVEAVKTYMGQRRISMLEEEEKKKMEEEMEQANAGWLTRTTSKLKTYVADVSEYLGSN(ph)SPRDPYLERQL

**Additional file 1: Figure S1. Protein sequences of selected MPK3/6 substrates.**

All SP and TP dipeptide motifs (minimal MPK phosphorylation consensus) are highlighted in yellow. Peptides recorded by mass spectrometry are underlined with lower case ph indicating phosphorylation of the preceding Ser or Thr residue (Hoehenwarter et al., 2013). Putative MAP-kinase docking domains matching the [K/R]-X_2-6_-[I/L]-X[I/L] motif are highlighted in magenta.

**Additional file 1: Table S1. Cloning Primers**

Primer name Sequence (5’-3’) Destination plasmid

MPK3 F BamHI GGATCCCCATGAACACCGGCGGTGGCC pGEX5x-3

MPK3 R EcoRI GAATTCCTAACCGTATGTTGGATTGAG pGEX5x-3

MPK4 F BamHI GGATCCCCATGTCGGCGGAGAGTTGTTTC pGEX5x-3

MPK4 R EcoRI GAATTCTCACACTGAGTCTTGAGGATTG pGEX5x-3

MPK6 F BamHI GGATCCCCATGGACGGTGGTTCAGGTCAAC pGEX5x-3

MPK6 R NotI gcggccgcCTATTGCTGATATTCTGGATTG pGEX5x-3

MKK1 F NotI GCGGCCGCATGAACAGAGGAAGCTTATGC pET_λ_HIS

MKK1 R XhoI CTCGAGGTTAGCAAGTGGGGGAATCAAAG pET_λ_HIS

MKK2 F EcoRI GAATTCGGCGCGCCACGAACCTCATGATC pET_λ_HIS

MKK2 R XhoI CTCGAGCACGGAGAACGTACCAGACAG pET_λ_HIS

MKK4 F EcoRI GAATTCATGAGACCGATTCAATCGCC pET_λ_HIS

MKK4 R XhoI CTCGAGTGTGGTTGGAGAAGAAGACG pET_λ_HIS

MKK5 F EcoRI GAATTCATGAAACCGATTCAATCTCC pET_λ_HIS

MKK5 R XhoI CTCGAGAGAGGCAGAAGGAAGAGGACG pET_λ_HIS

MKS1 F EcoRI GAATTCATGGATCCGTCGGAGTATTTTGCC pET_λ_HIS

MKS1 R SalI GTCGACATCTTGATCCCAAATATGACTAAAG pET_λ_HIS

VQ4 F HindIII AAGCTTTAATGGAGAATTCACCGAGATAC pET_λ_HIS

VQ4 R XhoI CTCGAGAGAAGTAGAAGCTGATGAAG pET_λ_HIS

AT1G78150 F EcoRI GAATTCATGGAGAGAAGTACTCC pET_λ_HIS

AT1G78150 R XhoI CTCGAGAACAAGTGCGATGCTTG pET_λ_HIS

AT1G78150 F ATGGAGAGAAGTACTCCGGTGAG pJET1.2

AT1G78150 R TTAAACAAGTGCGATGCTTGTC pJET1.2

AT2G26530 F ATGGAAGTGATGAGCTTGACTG pJET1.2

AT2G26530 R TTAGAAGGCGAATCGTCCTATTTG pJET1.2

AT3G11330 F ATGGCGGCAGAACCTAACCCTAAG pJET1.2

AT3G11330 R TCATAGCTGTCGTTCAAGGTAAG pJET1.2

AT4G38710 F ATGGAGAGAAGTACTCCGGTGAG pJET1.2

AT4G38710 R TTAGATTTTGTCCTCCGTCTC pJET1.2

**Additional file 1: Table S2. Mutagenesis Primers**

Primer name Sequence (5’-3’)

MPK3 F T119A cagttcagtgatgtttatatctctgctgaattaatggatactgatcttc

MPK3 R T119A gaagatcagtatccattaattcagcagagatataaacatcactgaactg

MPK3 F K67M K68R cgaacgagctagtagcgatgatgaggatagctaatgcttttgataa

MPK3 R K67M K68R ttatcaaaagcattagctatcctcatcatcgctactagctcgttcg

MPK4 F Y124A agagagagaacttcaatgatgtttacattgttgctgagcttatggacactg

MPK4 R Y124A cagtgtccataagctcagcaacaatgtaaacatcattgaagttctctctct

MPK4 F K72R ggagaggaggtagctatcaggaagattggtaatgctttt

MPK4 R K72R aaaagcattaccaatcttcctgatagctacctcctctcc

MPK6 F Y144A tttcaacgatgtttacatcgcggctgagttaatggacactgatctc

MPK6 R Y144A gagatcagtgtccattaactcagccgcgatgtaaacatcgttgaaa

MPK6 F K92M K93R actaacgagagcgttgcgattatgagaattgctaacgcttttgac

MPK6 R K92M K93R gtcaaaagcgttagcaattctcataatcgcaacgctctcgttagt

MKK1 F T218D S223D ggtgtcagcaagatcttgacaagcgatagtagtcttgctaatgatttcg tgGGCACATAC

MKK1 R T218D S224D GTATGTGCCcacgaaatcattagcaagactactatcgcttgtcaagatc ttgctgacacc

MKK2 F T229D T235D gtgagtaccgttatgacaaacgacgcaggtttagcaaacgattttgtggggactt acaattatatgt

MKK2 R T229D T235D acatataattgtaagtccccacaaaatcgtttgctaaacctgcgtcgtttgtcataacggtactcac

MKK4 F T224D S230D GTTAGTAGGATCTTGGCTCAGGATATGGATCCGTGTAATGATTCTGTTGGAACCATTGCTTAT

MKK4 R T224D S230D ATAAGCAATGGTTCCAACAGAATCATTACACGGATCCATATCCTGAGCCAAGATCCTACTAAC

MKK5 F T215D S221D TGGTGTGAGTAGGATCTTGGCACAAGACATGGATCCTTGTAATGACTCTGTTGGTACTATTGCTTATATGAG

MKK5 R T215D S221D CTCATATAAGCAATAGTACCAACAGAGTCATTACAAGGATCCATGTCTTGTGCCAAGATCCTACTCACACCA

AT1G78150 F S28A AAGTTCCACCGCCGGATGCTCCTTCCTCCGCTTCT

AT1G78150 R S28A AGAAGCGGAGGAAGGAGCATCCGGCGGTGGAACTT

AT2G26530 F S182A GCTCGATCACTTGCGCCTTTTCGGG

AT2G26530 R S182A CCCGAAAAGGCGCAAGTGATCGAGC

AT3G11330 F S439A CTCGGATCAAATGCTCCTCGAGACCC

AT3G11330 R S439A GGGTCTCGAGGAGCATTTGATCCGAG

AT4G38710 F S300A TCGTGAAGCCGGAGGCACCGGTCTTGGTCATT

AT4G38710 R S300A AATGACCAAGACCGGTGCCTCCGGCTTCACGA
